# The Odontological Society of Great Britain

**Published:** 1887-06

**Authors:** 


AMERICAN JOURNAL

—OF—

DENTAL SCIENCE.

VOL. XXI. THIRD SERIES—JUNE 1887. NO. 2.

ARTICLE I.

### THE ODONTOLOGICAL SOCIETY OF GREAT BRITAIN.

The usual Monthly Meeting of this Society took place on the 4th ult., Mr. Charles Tomes, F. R. S., President, in the chair.

A suggestion from the Council that Mr. Bland Sutton and Dr. Dudley Buxton should be elected Honorary Members was very cordially received.

The Curator announced several additions to the Museum, amongst them the skull of a young deer, in which the teeth and alveoli were much affected by disease.

Mr. Walter Harrison showed a model of an extreme case of double hair lip in a child five weeks old, operated on, with removal of the intermaxillary bones, by Dr. Whittle, of Brighton.

Mr. Penfold showed exact copies of instruments found at Pompeii, and preserved in the Museum at Naples, where they were labelled, "Dental Instruments," but they bore no resemblance to instruments now used by the profession, and Mr. Penfold was of opinion that they were intended for modelling clay with.

Dr. W. St. George Elliott showed a slight alteration in the Hodge Right Angle which he had made, with a view to overcome the tendency of the points to drop out. It consisted in attaching to the cover of the screw the lock-pin of the early S. S. White Right Angle, and the arrangement effected the desired result.

Mr. Ackery showed a pair of forceps designed by Mr. T. W. F. Rowney, of Derby, for dividing lower molars after their crowns had been removed. He also read notes of an obscure disease of the upper jaw in a medical student, aged 26, attended with numerous abscesses, which, after lasting five years, was cured by extraction of the first and second left upper molars, and carefully dressing the necrosed alveoli with aromatie sulphuric acid.

Mr. C. V. Cottrell showed models of the mouth of a youth aged 17 to 18, who had no illness from the time he was twelve months old. Previously to that he had had an attack of dysentery. His teeth were very yellow and perfectly soft, so much so that they could be cut with a knife. An uncle on the father's side had very similar teeth.

Mr. R. Hepburn had seen a similar discoloration result from the administration of acids.

Dr. Walker showed, on behalf of Mr. Humphreys, of Birmingham, the head of a calf with cleft palate extending from the inter-maxillary bone and palate portion of the superior maxillary bone. The two halves of the lower jaw were disunited, and the tongue was also bifurcated for some distance. The animal was otherwise strong and well.

### ON THE PATHOLOGY OF RIGGS' DISEASE, OR PYORRHŒA ALVEOLARIS.

By F. Newland Pedley, F. R. C. S., L. D. S. Eng.

We must all admit that it is a very unfortunate thing for a disease to be christened after the name of its discoverer, for there is always doubt whether his description was correct, and even where it is shown that the original interpretation was entirely wrong, the name may long survive to perpetuate blunder and bar the road to a more accurate perception.

How much greater does the difficulty become when a lesion is named after a man who never recorded a description of it.

In the year 1881, at the Medical Congress, Dr. Riggs denied that he had been personally instrumental in attaching his name to the complaint. But we are aware that Dr. Riggs invented a series of instruments for removing tartar buried beneath the gum, and hence the use of his name would wisely be confined to describe a form of treatment by scraping, directed against certain abnormal conditions of the gums, which he recognized as peculiar, but attributed to the effect of tartar.

Years went by and the designation Riggs' disease was retained, although it was used with quite a different meaning to the original, by many who applied it to a lesion of constitutional origin, and not necessarily associated with salivary calculus. To obviate this confusion, the appellation pyorrhœa alveolaris was substituted for Riggs' disease. This more recent name is not free from the objection of being a generic term merely indicating a symptom, and it requires a sharp definition to intimate in what sense we elect to adopt the title. For the general acceptance of the term pyorrhœa alveolaris, by dentists in Europe and abroad, assumes that we all recognize a certain definite disease to which it is applicable, and when a distinctive title is given to a complaint, a definition should be forthcoming, which in turn should be based on its pathology. Unhappily pyorrhœa alveolaris received a compromising name first, and still lacks a satisfactory pathology. In this dilemma we must, to-night, select the salient physical features and naked-eye appearances upon which we rely to distinguish this particular disease from others to which it bears points of resemblance, to do duty as a definition, and serve as a fixed point from which we can discuss and develop its pathology. Eventually I trust we shall be able to endow the lesion with a name of scientific import.

The local conditions to which the terms "pyorrhœa alveolaris" or Riggs' disease have been applied may be briefly quoted thus from the description of well-known authors. The mucous membrane, especially that adjacent to the teeth, appears deeply congested with venous blood, tumid and thickened, but not filling up their interstices at their necks, and detached for some distance from the surface, from the fangs. A thick fœtid discharge may often be pressed up between the teeth and mucous membrane which gives to the breath a very repulsive odour. This conditions of things continuing, the alveoli become absorbed, and at times more or less denuded, whilst the fangs of the teeth become coated with a layer of thin, hard, green-brown tartar. Ultimately, the disease progressing, the teeth one after another drop out. The alveolar margin is sometimes thickened in substance. The pain varies, it may be neuralgic from the first, but it is not generally considerable until the periosteum is fully involved.

Various theories have been advanced in explanation of the appearances and conditions occurring in the above complaint. I do not purpose going deeply into the germ theory. Doubtless numerous varieties of germs have been found in the purulent discharge from the alveoli, and an epidemic form of the disease is said to have appeared in Switzerland, and to have disappeared as rapidly as it came. The discharge contained peculiar micro-organisms, but the researches of modern bacteriology would not justify the conclusion that these germs were necessarily the cause of the disease; for even in the case of the well-known bacillus of tubercle, the question remains to be decided whether the bacillus is the cause or effect of the complaint.

American literature supplies us with a "catarrhal theory" for pyorrhœa alveolaris, based on the fact that the alveolodental membrane is continuous in structure with the deeper layer of the mucous membrane. It is suggested that catarrh of the mucous membrane of the mouth causes sloughing of the alveolodental membrane by cutting off the blood supply. A serious objection to this is that the chief vascular supply to the lining membrane of the alveolus is not derived from the mucous membrane of the mouth. We need also tarry little over another point, which consists in the statement that in some cases of pyorrhœa alveolaris pus was absent. Little importance need be attached to this, for the amount of pus formed is merely an index of the intensity of the inflammation, and the vascularity of the part. Chronic inflammation may well exist without pus, or the pus may be present, though invisible to naked-eye examination, or may be entirely absent at times, though present and visible at others.

The relation of salivary calculus to pyorrhœa alveolaris is a question of great moment, for, in the opinion of many dentists, tartar is the origin and essential cause of the complaint. This is obviously unsatisfactory as regards the ordinary crusts of calculus that are so commonly found clothing the crowns of teeth. All dentists see innumerable cases of this description, in which the gum and socket may recede as the result of the mechanical irritation from the calculus; but there is no considerable separation of the periosteum from the root of the tooth, and simple removal of the tartar and attention to cleanliness arrests the progress of the evil. But grave attention is frequently drawn to a thin layer of calculus that is found upon the roots of teeth, extending upwards beneath the inflamed periosteum of the tooth, and in some cases reaching the very apex of the root. The characters claimed as distinctive of this layer of deposit are that it is nodular, harder, and of a different colour to ordinary tartar; but it must be remembered that the calculus deposited beneath the periosteal pouches is subject to different conditions to that on the exposed portions of the tooth. Old tartar is often hard and nearly black in colour, and that which forms beneath the margin of the gum remains protected from removal by accident, mastication, or the use of a tooth-brush. Tartar mixed with pus is green. The nodular character of the deposit is probably attributable to the irregularity of the surface of the alveolus, and of the inflamed lining membrame, in contact with which the nodules are formed. The fact must not be disregarded that the deposit on the submerged roots is braided during its development with pus and effusions from the inflamed periosteum and gum. The difference in colour is not irreconcilable with the salivary origin of the deposit, when we remember how vastly tartar varies in shade under altered conditions, and in respect of its age; yet a formidable theory has been based chiefly on this variability of colour, which ascribes a "sanguinary" or "serous" origin to the nodular deposits that form beneath the margin of the gum.

Dr. Ingersol, an American dentist, discovered an "induration of granular formation" at the apex of a tooth that he had extracted, and he affirmed that he subsequently observed twenty such cases. Analysis showed dark colouring matter in these formations which, in his opinion, were only found in connection with ulceration, and this material he called "sanguinary calculus," because he inferred that it could not have come from the saliva.

To establish his point it would be necessary to prove that these calcareous deposits were entirely inaccessible to saliva. It is an eloquent coincidence that the analysis only differed from salivary calculus in the presence of colouring matter, and, granting for the moment that this were effused as some form of hæmoglobin from the capillaries of the inflamed periosteum, it would not necessarily follow that the calcareous deposits came from the same source. None would be prepared to express surprise that an irritated periosteum should occasionally deposit on the root of a tooth small masses of calcareous material, similar in nature to callus or osteophytes. In the vast majority of instances the nodular deposit on the roots of teeth is plainly observed to progress in a direction from the cervix towards the apex, and not the reverse. It may here be mentioned that tartar has never been seen upon the surface of an unerupted tooth.

One other form of salivary calculus should be referred to, and that is the annular collection of hard dark material that is so often found beneath the free edge of the gum. Its formation and longevity are favoured by the protection of the margin of the gum, even in the mouths of many patients who give scrupulous attention to the use of the tooth-brush. Hard food and careful brushing may remove every vestige of tartar from the exposed portions of the teeth without being able to prevent the formation of a ring of calculus beneath the shelter of a gum margin that may have been congested from one of many causes.

In some cases of pyorrhœa alveolaris there has been no tartar whatever found on the roots of teeth extracted, but unfortunately this is not a final argument, for it may be legitimately urged that a thin layer of tartar might have been present originally, but was dissolved away by the vitiated secretions of the mouth, which had undergone a change in character and chemical reaction.

The presence of salivary calculus is insufficient in itself to account for a special disease with features of constitutional origin, and there is ample reason for seeking the cause of pyorrhœa alveolaris in a systemic condition. It is known to occur in the mouths of patients whose health has been undermined by debilitating influences and injudicious habits of living. Frequently the disease is seen to attack the opposite sides of the mouth in a symmetrical manner. This is opposed to the assumption of a local origin of the affection. Cases have followed the prolonged use of mercury, iodide of potassium, or chloride of sodium, though it is open to doubt how far the blame should rest on the complaints for which these drugs were taken. Pyorrhœa alveolaris is a common sequel of malarial fever in America, and these patients are said to experience a great craving for salt.

Young patients recovering from eruptive fevers are sometimes subjects of pyorrhœa alveolaris. Frequent pregnancies are a rife source of the disorder.

The recent researches of Dr. Bland Sutton in Comparative Dental Pathology have supplied us with invaluable data. He established the fact that animals kept in captivity suffered from premature loss of teeth. Mr. Sutton collected numerous specimens of recession ot the alveolar process, both when tartar was present and when it was not. At a meeting of the Pathological Society he showed a considerable series of the skulls of monkeys whose jaws were affected with recession of alveolus as a result of rachitis, and he recorded a similar condition occuring in a case of mollities ossium. During the time I was Dental Surgeon to Evelina Hospital, and since, I have seen many cases of premature loss of teeth from recession of alveolar process and gum in rachitic children. Mr. Bland Sutton showed that, in the case of lions kept in captivity, unsuitable food, by preventing the assimilation of lime salts, produced cleft palate in the progeny. Also, that a series of pregnancies at short intervals in the case of a female dog led to similar defects in the osseous system of the offspring by exhausting the maternal store of lime salts. Also, that in a snake kept in captivity the bone of attachment becomes absorbed, leading to the loss of the teeth, which were abnormal in size, number and attachment. The consideration of the above three facts in connection with one another offer a striking parallel to the hereditary form of pyorrhœa alveolaris, and suggest that an element of the complaint might be hereditary defects in the structure of the teeth and alveolar process.

Here one may cite the experience of all dentists that the removal of affected teeth usually cures the lesion where the teeth have stood, and that the disease has a tendency to spread from affected teeth to those in their neighbourhood.

Attention has recently been drawn to tabes dorsalis in connection with the premature shedding of teeth. I

recently examined two cases of advanced tabes dorsalis in the wards of Guy's Hospital. The disease was well marked and neither patient had ever used a tooth-brush, but there was no evidence of pyorrhœa alveolaris. All wasting diseases and depressed conditions of the nervous system are conducive to the premature shedding of teeth, but pyorrhœa alveolaris is not a necessary concomitant of advanced tabes dorsalis. My friend Dr. Hale White is present to-night, and I leave this phase of the subject in his able hands.

Whilst a depressed condition of the constitutional strength is the most important factor in determining the diseased pyorrhœa alveolaris, local causes favour its development, and in patients of this systemic tendency the outset of the attack may be traceable to some slight influence, such as the use of a very hard tooth-brush, coarse tooth-powder, or the like. A crowded arrangement of the teeth may occasion it, or one tooth may be so placed that the mucous membrane around it escapes healthly friction and becomes the seat of the mischief. No doubt the subsequent deposit of tartar on the denuded portion of the root aids as a foreign body in keeping up the irritation of the periosteum.

One gathers from the somewhat diffuse literature of this subject a consensus of opinion that the early stage of pyorrhœa alveolaris is characterized by an hypertrophied condition of the muco-periosteal fold around the teeth, accompanied by dilatation of capillary loops, enlargement of papillæ, and rapid proliferation of epithelial cells. Later on the gum becomes firm and contracted, and displays increase of fibrous tissue. What changes go on in the socket during the recession of the alveolar margin I am unable to state, for the simple reason that I cannot get a recent specimen. The theory seems to be wide-spread that the inflamed periosteum becomes separated from the subjacent alveolar margin, thus depriving it of its vascular supply, and leaving it denuded, rough and carious. This may be so in some cases, but there are many in which the alveolar margin recedes and yet the result of probing is opposed to the supposition that the bone is bare. In these instances the socket wastes without becoming denuded of its periosteum.

At the Medical Congress in 1881, Dr. Walker opened a discussion on Riggs' disease and exhibited a number of microscopic specimens, the conclusions he arrived at being that in the present state of pathological knowledge no distinction could be traced between the loss of a tooth socket prematurely by disease, its absorption after extraction or its wastings in old age, the microscopic characters being identical, viz.:—loss of vascularity, increase of fibrous tissue, subacute inflammation passing into the depths of the alveolar processes adjacent to the inflamed gum. The report does not mention to what extent this hypothesis was accepted, and the microscopic appearances described are unsatisfactory in this respect, that the section that would represent pyorrhœa alveolaris was taken from the mouth of an aged dog whose teeth were loosening.

The Museum of the Odontological Society possesses specimens presented by Mr. Bland Sutton of the skulls of animals which died in captivity, and whose jaws show evidence of a disease which seems to be an analogue of pyorrhœa alveolaris in the human subject. One of these, the skull of a carnivor, is very striking. There is not an even recession of the bone from the margin towards the apex, but the alveolus is obviously attacked to some distance from the margin. Where teeth have recently been lost, the surface of the bone shows depressions and hollows, irregular in outline, with eroded patches and a few spicules of bone that are apparently of new formation. Where the destructive process is at its full activity around a tooth that is being lost, a wide space is noticed to have formed between the tooth and the jaw, by absorption of the bone and the roots of the tooth. The space between the tooth and the jaw is as great at the apex of the root as at the neck. Around the affected tooth the alveolus is lost on the buccal surface, and wherever the root is laid bare it is coated with greenish-brown tartar, but the destructive process is seen to be going on in places where no tartar is present. There is a very peculiar appearance about the alveolar septum between the roots of the teeth, at those parts where it is thickest and most vascular. Here one can trace the outline of what its shape has been, but the bone has been reduced to a condition suggestive of filligree or trellis work, and the last support of the loosening tooth is obviously pedicles of alveolar process with efflorescent extremities of sponge-like bone. The cementum on the root of the teeth is thinned, and the root itself is greatly truncated by absorption. The general impression remaining is that one is examining a case of osteitis extending from the margin of the alveolar process to a point some little distance beyond the level of the roots of the teeth, and by no means going hand in hand with the deposit of tartar.

The result of local treatment aids us by demonstrating the very refractory nature of well-marked cases. The thorough removal of any tartar from the roots of affected teeth by means of instruments and chemical reagents, and the subsequent use of astringents, gives good results, at least temporarily, by allowing the gum to more closely embrace the roots of the teeth, and preventing the accumulation of tartar and discharge in the alveolar pockets; but relapses are common, and our main attention must be directed to general constitutional treatment, which comes within the domain of the physician.

The inferences that follow from the points I have enumerated lead to the assumption that the affection which to-night we are calling pyorrhœa alveolaris is essentially of constitutional origin. In man and in lower animals it is found connected with wasting diseases and depressed conditions of the system. The local exciting cause may be of a very trivial nature.

The weight of evidence tends to place pyorrhœa alveolaris in the category of bone diseases. The exposed position of the alveolar margin and its intimate relation with organs of such feeble vascularity as the teeth, go far to explain why it is this portion of the alveolus that is first affected, and also the usual arrest of the disease by the removal of the teeth.

### RECENT RESEARCHES ON SYMPTOMATIC ALVEOLAR ARTHRITIS.

BY DR. E. MAGITOT.

Having referred to the history of and the literature on the disease, the writer says these lesions consist, in fact, of a derangement of an inflammatory nature, affecting at the same time the tissue which has been called the *alveolodental periosteum*, and the bony covering which is subjacent to it, viz., the cement. The term osteoperiostitis was thus justified by the simultaneous lesion of a periosteal and osseous layer. The expression conformed, moreover, to the rule adopted in France on the subject of medical nosology, which gives to every disease a name corresponding to the anatomical lesions which characterize it.

Be that as it may, this last description does not appear to have satisfied all authors, for several of them have gone back to the term *expulsive gingivitis*, which is evidently incorrect, since the gum is never primarily attacked. Others, again, have taken up the term *alveolar pyorrhœa*, which term seems to prevail amongst English practitioners, and particularly with our colleague, Mr. F. Newland Pedley; whilst others, Dr. David, of Paris, for example, propose to call it Fauchard's disease (*Maladie de Fauchard*), after the name of the author, who was one of the first to mention it as described. Following the same idea it is called in England and America Riggs' disease.

If this tendency to multiply without any definite rule the names of the same disease be persevered in much longer, one calling it from one of its symptoms, *pyorrhœa* or *suppuration*, others giving to it the name of an author who has described it with more or less exactitude, extreme confusion will be the result.

Moreover, amongst this diversity of titles the rule, referred to above, of invariably taking as the basis of nomenclature the pathological anatomy of disease, is completely forgotten.

Thus, while regretting to add in our turn a new name to a nomenclature already so long, we feel ourselves, nevertheless, obliged to substitute once more for the designation hitherto employed a title which, faithfully conforming to the precise rules indicated, will establish the nature of the disease with all the exactitude desirable. This title is that of *Symptomatic Alveolar Arthritis.*

The justification of this choice is very simple. The recent researches made in France, particularly those by Mons. Malossez, have clearly established the fact that that which we have described as *periosteum* around the roots of the teeth ought to be considered, not as a membrane, distinct and capable of being dissected off, like the osseous periosteum, but rather as a true ligament.

This manner of looking at it, already indicated by Kolliker in Germany, and by Ranvier in France, in his course of lectures at the College of France, is generally adopted in our country. We, ourselves, are in complete harmony with it, thus accepting the necessity of modifying in our own studies the interpretation which we had, until now, assigned to this part of the dental organ.

The teeth are then in reality articulated with the jaws by the intermediary of a *ligament*, and every lesion of such an articulation ought to be assigned to the category of *arthrites.* We shall, then, henceforth thus describe the affection with which we are dealing.

As to the term *symptomatic*, we are induced to avail ourselves of it for the reason that this form of disease ought to be carefully distinguished from the affection hitherto described under the name of *alveolodental periostitis*, and to which henceforth the name *simple alveolar arthritis* will be most applicable. This latter, which is in fact sometimes spontaneous or traumatic, but more often the result of the last stages of caries, possesses a character and progress quite distinct from *symptomatic alveolar arthritis*. While the last commences at the neck of the root, the former appears only at the apex. Moreover, the loosening and displacement of the teeth, the initial phenomena in the symptomatic form, are very rarely met with in the simple form. Finally, suppuration of the alveoli does not exist in the latter. Let us add that the word *symptomatic* well expresses the relation which this arthritis holds with regard to the general diseases or diatheses which are invariably the first cause of it. These different specific terms indicate clearly the differential signs of the two affections, which can thus preserve the generic denomination of arthritis, the term *symptomatic* sufficing, in our opinion, to distinguish that with which we are occupied in this work from that of *simple alveolar arthritis*, which name should remain attached to the ordinary form.

As we have already indicated in our previous researches, the causes of this disease should be looked for, not in the local conditions of the mouth or gums, but in certain conditions of the general health. The disease ordinarily attacks a single tooth; occasionally several teeth may become involved, but in the latter case the affected teeth are not necessarily contiguous, they may occupy different positions in the mouth at a distance from each other. Toirac and M. Oudet believe they have observed that the inferior incisors were more particularly the seat. We have not ourselves recognized this peculiarity, which appears to us to belong more especially to gingivitis, which may in certain cases be confounded with symptomatic alveolar arthritis.

The teeth most frequently affected are in order of sequence : firstly, the molars, then the inferior incisors, the bicuspids, the superior incisors, and lastly the canines. We have never observed this disease affecting simultaneously the whole of the teeth. At one time it is situated on one or two inferior or superior incisors, at another the incisors are spared, and one or more molars are affected, ordinarily two or three on different sides of the mouth. Sometimes the disease only attacks a single root of these latter, or it may be only a single side of a root, a circumstance which preserves to the root a certain degree of solidity for a considerable time. The teeth affected with symptomatic alveolar arthritis do not generally present any preceding or accompanying alteration. Caries, for instance, has no connection with this disease, and if this complication is present it is simply an accidental occurrence. It is, indeed, worthy of remark that the local conditions which accompany the development of symptomatic alveolar arthritis appear to be just the opposite to those which accompany the production of caries; the buccal saliva is in fact rather alkaline than acid, an accumulation of tartar more or less abundant is observed in the places where it is usually met with. One might be tempted on the first glance to attribute to this deposit a part more or less active in the etiology of this disease, but it is not so. The deposit of tartar is a secondary matter, and in all cases, its formation being generally uniform and continuous in the same region, it cannot be cited in the production of an isolated and local affection.

It is very important to note the various conditions presented by those who are the subjects of symptomatic alveolar arthritis. The age at which this affection is generally observed is not either in youth or in advanced age, but between the thirtieth and fiftieth year of age; it is equally frequent amongst men and women, and amongst the latter it appears often accompanied with the complex phenomena of the "menopause." In a certain number of cases symptomatic alveolar arthritis supervenes during a state of perfect health, and whatever pains we may take to find out the cause, we cannot recognise it either in the local conditions of the mouth, or in any disorder of the economy; nevertheless, the temperaments which appear predisposed to it are almost exclusively of the sanguine and bilious varieties; constitutions appearing in other respects vigorous, yet subject to cephalic congestion. Persons of sedentary occupations, those who are engaged in an office, are particularly predisposed to it. We have already mentioned several times the relationship produced by the appearance of the crisis attending the cessation of the menstrual discharge, or of the hæmorrhoidal flux. This influence of temperament has again a close connection with heredity, which in certain families has appeared to us to influence the appearance of the disease; thus it has made its appearance in members of the same family during two or three generations, and in analogous conditions of age and constitution.

Certain intestinal phenomena are observed, whether they be coincidences or whether of etiological connection. Habitual constipation is met with in the subjects attacked, and one of the physicians to the Paris hospitals, M. Fidal, has observed that they often present dyspeptic phenomena. Perhaps these latter may have been due to difficulties in mastication; in any case, we have personally had the opportunity of verifying this assertion. Some general or diathetic conditions exercise a considerable effect on the production of this disease. Thus eruptive fevers have, as is known, sometimes caused a falling out of the teeth, a result produced by symptomatic alveolar arthritis.

We recently observed in a little girl seven and a-half years old alveolar arthritis following a severe attack of whooping cough. The temporary teeth, and above all the permanent teeth already erupted, were surrounded by pads of inflamed gum, with alveolar suppuration and lossening of the teeth (one out of the first permanent molars had already fallen out, and another was greatly threatened.) The affection appeared to consist in an ulcerative stomatitis and a simultaneous symptomatic alveolar arithritis.

Gouty and rheumatic subjects often present this kind of arthritis, as also do individuals attacked with anæmia caused by long illness; but there are no general lesions which exercise a graver influence on the production of this disease than those of albuminuria and, above all, diabetes. As regards the first we are dealing here, as is well understood, not with symptomatic albuminuria, but with what is properly called Bright's disease.

In glucosuria this phenomenon is absolutely constant, and even constitutes one of the primordial signs of the morbid conditions. In fact it is mostly found in the descriptions of authors that at the commencement of diabetes the teeth become loose and carious. This assertion concerning caries is not correct, but the first is perfectly so, and corresponds to the symptomatic alveolar arthritis which follows in its development the same course and the same progression as the general disease, causing at its termination the loss of a considerable number or the whole of the teeth. Such are the results of our researches, which we communicated to the Academy of Medicine, under the name of "A Treatise on the Value of Diagnosis in Saccharine Diabetes of Alveolodental Osteoperiostitis."

We have not recognized in any way that any other conditions of the health were in connection with symptomatic alveolar arthritis. Thus certain diathesis, such as syphilis, in which the tertiary symptoms affect the bones and fibrous tissues; do not appear to produce it; mercurialization and gingivitis, however great its severity, do not appear to become either the principal or even the occasional cause.

We do not wish here to enter fully into all the considerations concerning the treatment of symptomatic alveolar arthritis. We have detailed them to a considerable extent in our previous publications, but we should like to particularly insist on the value of the action of a certain class of remedies which enter into antiseptic medicine,

We are in fact confronted with a septic and contagious disease, of which the essential symptoms are an abundant alveolar suppuration, loosening and displacement of the teeth, recession of the gums—in a word, all the conditions suitable to make of this lesion a spot favourable for the action of septic agents.

These agents undoubtedly exist in this disease. The microscopic preparations of pus proceeding from alveolar diseases have betrayed the existence of a large number of organisms of very diverse forms, amongst which it is, if the truth be spoken, still difficult to distinguish the variety which ought to be regarded as the essential and exclusive morbid agent of this disease. Further researches, we are convinced, will enable us to realize the culture of the pathogenic agent peculiar to symptomatic alveolar arthritis.

Some interesting experiments, undertaken by MM. Malassez and Galippe, for this purpose have already put beyond doubt the parasitic nature of this disease, and it is also confirmed by the frequent propagation of the disease from one alveolus to another in the same mouth, as well as by contagion from one individual to another, as observed by different authors and ourselves. All that we now wish to insist upon is the value of antiseptics in the treatment of this disease. With this view we would recommend such applications as alcohol, carbolic acid, per-chloride of mercury, &c. For this purpose we have selected the following formulæ:—

| | |
|---|---|
| No. 1—Hydr. perchlor. | 50 c g. |
| Aq. Distil. | 1,000 grms. |
| No. 2—Acid carbolic crystal } | — |
| Ether } | aa 5 " |
| Alcohol | 10 " |
| No. 3—Acid borac. | 10 " |
| Aq. Distil. | 500 " |
| Alcohol | 50 " |

To these formulæ we could have very well added others by employing other antiseptics, such as the permanganates, salicylates, salicylic acid, &c. It will be necessary besides to have recourse to the employment of medicines which will modify the local condition, as well as to attend to the treatment of the general health, paying due regard to the diatheses which influence the disease.

Thus the treatment of symptomatic alveolar arthritis can be summed up in the three following paragraphs :—

1st. To render antiseptic the alveolus, the seat of the arthritis.

2nd. To modify the condition of the affected tissues by the application of astringents and caustics.

3rd. To treat the general conditions or diatheses which govern the local manifestations.

Concerning the second point—that is to say, the local treatment of the affected tissues—it is known that we have advocated applications of chromic acid, employed chemically pure and monohydrate, as a powerful caustic susceptible of acting on the vitality of the tissues of the gum, and above all, on the dental ligament. Let us add that chromic acid may also be considered at least equal as an antiseptic to any of the drugs usually employed in that sense. We can mention again, among the modifying agents, pure carbolic acid, chloride of zinc, and lastly heat applied by means of the galvanic cautery.

Finally, as concerns the diatheses, the dominating influence of which we have already referred to, we shall not here indicate the treatment so as not to exceed the limits of this paper.

From the preceding considerations we believe we may deduce the following conclusions:—

1st. The affection characterized by alveolar suppuration, by the loosening and falling of the teeth, ought to be considered as a true symptomatic alveolar arthritis, septic and contagious, and which ought henceforth to remain known by that name in surgical nosography.

2nd. This affection is most often met with under the influence of certain general conditions and diatheses, and as secondary to eruptive fevers, &c., of which it may form either a complication or a sequela.

3rd. The therapeutics of symptomatic alveolar arthritis ought to consist essentially in the employment of antiseptics, of local alteratives, astringents, or caustics, without prejudice to the treatment of the general conditions which are its determining causes.

DISCUSSION.

The President remarked that the term "arthritis," used by Dr. Magitot, would probably sound strange to most of those present, though it did not really mean anything very different from what they were accustomed to. It was based on the opinion, held by the author of the paper and others, that what was generally known in England as "the alveolodental membrane" was, properly speaking, a ligament, and that inflammation of it should not, therefore, be referred to as *periostitis*, but as *arthritis*. This view of the nature of the alveolo-dental membrane was founded, in part, on Comparative Anatomy.

Dr. Hale White said he had been interested for some time past on the subject under discussion, having had his attention called to it by the premature loss of the teeth which occurred in cases of tabes dorsalis. He showed a tooth which had fallen from the mouth of a patient affected with this disease. It was a lower molar; there was a little tartar on it, but its loss had been unaccompanied by any signs of inflammation. This falling of the teeth was, according to some authors, a rare but genuine symptom of tabes dorsalis, and there were apparently two ways in which it could occur. The soft structures around the jaw might swell, and one by one the teeth might fall out, although themselves quite sound; this might be followed by some necrosis of the jaw with discharge of a sequestrum. Or the teeth might fall out without any apparent cause, and the patient might lose a number of sound teeth within a few days without any pain or swelling of the gums. Richardieu has recorded one example of the first kind, whilst examples of the second had been recorded by Hoffman, Lewis, Vallin, Demange, Gowers and Fere. In many of the cases it was an early symptom, but still it was a rare one; indeed, it was a question on which the dentist might be able to give the general physician some information, whether the teeth fell out more commonly in persons suffering from tabes dorsalis than in persons not so affected. He (Dr. White)

was inclined to think that the teeth might fall out in many diseases, and that such an occurrence should not be exalted to the position of a symptom peculiar to tabes dorsalis.

Dr. White next referred to the atrophy of the lower jaw, described by Fere, as sometimes accompanying tabes dorsalis, and inquired whether dentists had met with such a condition of jaw. Disease of the tempero-maxillary articulation had also been described and regarded as significant of this disease, but it seemed probable that this also was only a coincidence, and that it was really caused by rheumatoid arthritis.

He should, further, be glad to know whether any examples of inco-ordination of the muscles of mastication had ever been observed by any of the members, and was the use of the "jaw jerk" of any advantage?

In trying to trace the cause and effect in these cases, it must be remembered that any wasting disease would affect the teeth also, whilst on the other hand any difficulty in mastication would lead to impaired nutrition.

Mr. J. Bland Sutton, in reply to an invitation from the President, said he had come to learn and not to speak. He could only endorse the views which Mr. Pedley had expressed in his paper. The disease was undoubtedly chiefly of constitutional origin, but it also required local treatment; and as the physician did not consider that the teeth were in his province, and the dentist thought the constitution was not in his, the treatment of this disease fell between two stools. He had examined a large number of cases of rheumatoid arthritis, and found that the premature loss of the teeth was a very common feature. It was also met with in mollities ossium and in other wasting diseases. As to tabes dorsalis, it was, he believed, only a name for a group of symptoms not connected with any definite pathology. It was, however, a curious fact that some of the carnivora got symptoms closely resembling those of the locomotor ataxy in the human subject, including the falling out of the teeth and absorption of the alveoli.

To arrive at a proper understanding of Riggs' disease it was necessary not only to look in the patient's mouth, but also to note carefully the constitutional state, and especially any indications of the presence of rheumatoid arthritis, and to obtain particulars of the patient's family history. It was only by the careful collection of facts that it could be ascertained whether Riggs' disease was really a disease, or whether the name was merely a convenient cloak for ignorance.

Mr. F. J. Bennett said Mr. Pedley appeared to consider the vascular supply to the periosteum was derived principally from the vessels of the pulp and of the alveolus. But it was a fact which could be clearly seen from specimens in the Museum that there was a very free supply from the submucous tissue of the gum. The capillary network where the gum joined the neck of the tooth was very peculiar from the looped arrangement of its vessels, a description and illustration of which was given in Salter's "Dental Pathology," and in Tomes' "Dental Surgery" there was a figure of an injected specimen showing the vessels much enlarged in a monkey.

Tracing the course of the disease, it would be found that the first symptom was increased vascularity at the gingival margin and separation of the gum from the neck of the tooth; that there was a strong tendency for this part to become congested, and that this congestion was followed by effusion into the submucous tissue, causing gradual destruction of the fibrous attachment of the periosteum of the tooth.

The belief in the dependence of Riggs' disease upon tartar appeared to be still very general, but a strong argument against this was that cases were frequently met with in which persons had large accumulations of tartar which had been forming for years, but yet had not caused separation of the gum from the tooth. This, he thought, was quite sufficient to show that there was no necessary connection between the presence of tartar and Riggs' disease.

He agreed with Mr. Pedley that the disease was predisposed to by causes which brought about a lowered state of constitution, as anæmia, dysentery, frequent pregnancies, feeble circulation, &c.

Mr. S. J. Hutchinson said he wished to say a word in defence of the name by which the disease which was the subject of discussion had been generally known for some years past. He was quite aware that Dr. Riggs was not the first to recognize the disease, but that the symptoms were accurately described in the first edition of Tomes' "Dental Surgery." But Dr. Riggs was the first to point out that the removal of the free edge of the alveolus had the effect of checking to a considerable extent the progress of the disease. He had in fact pointed out the only method of treatment which had hitherto proved successful, and he (Mr. Hutchinson) thought that the connection of Dr. Riggs' name with the disease was not an undeserved compliment. It must be admitted that the profession was still in darkness as to the origin of the disease, and further investigations into its etiology and pathology were greatly needed.

Dr. Walker said he should be pleased to present to the Society the sections (eighteen in number) which had been prepared for examination by the members of the International Medical Congress of 1881 by Dr. Gibbes, and to which Mr. Pedley had referred in the course of his paper. The members could then examine them at their leisure, and form their own opinions respecting them.

Mr. Moore said it had been stated that evening that Riggs' disease never affected all the teeth. With reference to this point he would mention the case of a lady, aged thirty, whom he had treated for this disease about fifteen years ago. All her teeth were loose, and covered with hard nodular tartar, from the crown to the apex of the root. Various remedial measures were tried, but proved useless, and eventually all her teeth had to be extracted.

The President said that some years ago, when on a visit to the States, he saw Dr. Riggs carry out his treatment in a good many cases. He did not remove the edge of the alveolus, for this was already gone before the cases came under treatment, but he gouged out and scraped away all the softened bone surrounding the affected teeth. His treatment was somewhat severe, but its *immediate* effects were certainly very good. In all the cases, however, which he (Mr. Tomes) had been able to trace out afterwards, the disease had recurred and run its usual course.

The question with reference to the connection of tartar with the disease, which had been referred to by several speakers during the discussion, received, he thought, some elucidation from a specimen in the Museum. On looking at this it would be seen that the ring of hard tartar surrounded the tooth at some distance from the edge of the alveolus, and that the surface of the tooth between these points was quite clean. It seemed evident from this that the tartar could not be the cause of the absorption of the bone.

With reference to what had been said about the premature loss of teeth by patients suffering from locomotor ataxy, he could only say that in two cases of this disease which had come under his notice the teeth were in a perfectly healthy state. In both cases the disease was at a comparatively early stage.

Mr. Heth said the majority of those who had taken part in the discussion appeared to agree in considering that Riggs' disease was of constitutional origin, and he thought hardly enough attention had been paid to its local causes. It appeared to him that a good deal might be said in opposition to the theory of its being a constitutional disease. In the first place many of those who suffered from pyorrhœa alveolaris were in what might be termed vulgar good health. Then it was not unusual to find a single tooth affected in the upper or lower jaw, and sometimes only a single root, as the palatine root of an upper molar, the other being healthy. The way in which the disease spread on either side of an infected centre was much more suggestive of a local than of a general origin. The effect of local remedies also served to strengthen this view, and the fact that the disease could not be checked, if not cured, by antiseptics. He thought that the difficulty in effecting a permanent cure arose from the fact that sufficient attention was not paid to the disease in its early stages.

Dr. Field said he could not elucidate the pathology of the disease, but he should be glad if he might be allowed to say a few words with regard to some practical points in connection with it. He would repeat what he had stated before the Society not long since, that cases of genuine Riggs' disease were comparatively rare, and he doubted if one had ever been cured. He knew that Dr. Riggs and others asserted that they had cured cases, but he himself had never yet seen a patient permanently cured. The disease was invariably connected with a depressed state of the nervous system. In several of the cases he had seen the patient was suffering from Bright's disease, and one was suffering from locomotor ataxy. Medical and dental treatment must go hand in hand. Locally he still used Dr. Riggs' instruments, carefully removing all tartar and then applying aromatic sulphuric acid. The gums should at the same time be stimulated by rubbing and by massage with the fingers. By these means good results could be obtained for the time, and the progress of the disease checked, but he almost despaired of obtaining a permanent cure.

Dr. Geo. Cunningham thought that if Dr. Field would make further inquiries he might be induced to modify his opinions. It was Dr. Mills, of Brooklyn, who was chiefly instrumental in getting Dr. Riggs' name attached to the disease. He himself suffered from it, and was cured by Dr. Riggs. Dr. Riggs was a very modest man, and no reflections could be thrown upon him for the use which had been made of his name.

It was certainly very desirable that more exact knowledge should be obtained with reference to the pathology of this disease, and he would suggest that if dental students would examine the mouths of all the cases brought to the *post-mortem* room they would be sure to come upon examples of Riggs' disease, and might obtain sections which would be far more reliable than any which could be got from animals.

Mr. Storer Bennett said he should like to call the attention of members to the fact that there were a number of very interesting specimens of premature absorption of the alveoli to be seen in the Museum, and he thought that if members would take the trouble to investigate them carefully, they might add a good deal to their knowledge of the subject.

Mr. Pedley, in reply, said he could not agree with Dr. Magitot in thinking that the disease was contagious. He had never heard of a dentist getting it from a patient, and as to its spreading to contagious teeth, it spread, not by contagion, but by continuity of tissue. As to the germ theory, this had been frequently suggested, but never proved. No doubt there was an abundance of germs present, but it could not be proved that any of these were specific. In the discussion which took place on this subject at the International Medical Congress of 1881, Dr. Arkovy announced that he had discovered and cultivated some organisms which he thought were peculiar to this disease, but these observations had never been confirmed, and Dr. Arkovy himself did not appear to have thrown any further light on the subject.

Dr. Magitot also denied that syphilis or mercurial treatment predisposed to pyorrhœa alveolaris. He (Mr. Pedley) could only say that this did not accord with his experience.

So far as his limited experience enabled him to judge, he thought Dr. Hale White was right in concluding that there was no direct connection between Riggs' disease and tabes dorsalis. Certainly in the cases of this disease which he had examined at Guy's Hospital the gums were quite healthy.

He was not surprised to hear from Mr. Sutton that patients who suffered from osteo-arthritis were liable to suffer also from pyorrhœa alveolaris; it only confirmed his experience that it was a frequent complication of all chronic diseases resulting from constitutional debility.

Mr. F. J. Bennett supported the "catarrhal theory." Some cases of congested gum margin might be explained in the way he had suggested, but his theory certainly would not account for the course and symptoms of pyorrhœa alveolaris.

Dr. Walker had kindly offered to present his series of specimens to the Society. He (Mr. Pedley) readily admitted that several of these were valuable and instructive but he objected to the sections taken from the jaw of an old dog which was losing its teeth being regarded as typical of Riggs' disease.

He could not quite understand the grounds on which Mr. Hutchinson advocated the retention of the name "Riggs' disease." He gathered that Mr. Hutchinson wished to retain it because it commemorated a method of treatment which was based on a theory which was now known to be incorrect, and which had itself proved unsatisfactory in practice.

He congratulated Dr. Field on having changed his opinions since 1877, and come round to what he believed to be a truer view of the case.

Dr. Cunningham had alluded to what he said was "a genuine case of Riggs' disease," but as there was no definition of Riggs' disease to be obtained, it was impossible to say exactly what was meant by "a genuine case." Dr. Riggs attributed the disease to the effects of tartar, and his treatment was intended to effect its removal. There was no doubt that the treatment did do good for a time, but only by the removal of *debris*, and to deal thus roughly with diseased bone, breaking and lacerating it with instruments, appeared to him on the face of it an unwise course to pursue.

Mr. Tomes had referred to a specimen in the Museum, which showed clearly that tartar was not to be found where the destruction of bone was going on most actively. He (Mr. Pedley) had alluded to this in his paper, and he had no doubt had the same specimen in mind.

Mr. Hern appeared to think that a ring of hard dark tartar was the distinguishing characteristic of Riggs' disease. What then would he call the cases in which there was no tartar to be found, but which were identical in every other respect?

After the usual votes of thanks to the contributors of casual communications and the authors of the papers read during the evening, the Society adjourned.—*London Dental Record.*

